# Major QTLs, *qARO1* and *qARO9*, Additively Regulate Adaxial Leaf Rolling in Rice

**DOI:** 10.3389/fpls.2021.626523

**Published:** 2021-02-19

**Authors:** Su Jang, Sangrea Shim, Yoon Kyung Lee, Dongryung Lee, Hee-Jong Koh

**Affiliations:** ^1^Department of Plant Science, Plant Genomics and Breeding Institute, Research Institute for Agriculture and Life Sciences, Seoul National University, Seoul, South Korea; ^2^Department of Chemistry, Plant Genomics and Breeding Institute, Seoul National University, Seoul, South Korea; ^3^King Abdullah University of Science and Technology (KAUST), Division of Biological and Environmental Sciences and Engineering (BESE), Thuwal, Saudi Arabia

**Keywords:** leaf rolling, ideotype breeding, rice (*Oryza sativa* L.), quantitative trait loci, ideal plant type, v-shape leaf

## Abstract

Moderate leaf rolling is considered optimal for the ideal plant type in rice (*Oryza sativa* L.), as it improves photosynthetic efficiency and, consequently, grain yield. Determining the genetic basis of leaf rolling *via* the identification of quantitative trait loci (QTLs) could facilitate the development of high-yielding varieties. In this study, we identified three stable rice QTLs, *qARO1*, *qARO5*, and *qARO9*, which control adaxial leaf rolling in a recombinant inbred line (RIL) population derived from a cross between Tong 88-7 (T887) and Milyang 23 (M23), using high-density SNP markers. These QTLs controlled the rolling phenotype of both the flag leaf (FL) and secondary leaf (SL), and different allelic combinations of these QTLs led to a wide variation in the degree of leaf rolling. Additive gene actions of *qARO1* and *qARO9* on leaf rolling were observed in a backcross population. In addition, *qARO1* (markers: 01id4854718 and 01asp4916781) and *qARO9* (markers: 09id19650402 and 09id19740436) were successfully fine-mapped to approximately 60- and 90-kb intervals on chromosomes 1 and 9, respectively. Histological analysis of near-isogenic lines (NILs) revealed that *qARO1* influences leaf thickness across the small vein, and *qARO9* affects leaf thickness in the entire leaf and bulliform cell area, thus leading to adaxial leaf rolling. The results of this study advance our understanding of the genetic and molecular bases of adaxial leaf rolling, and this information can be used for the development of rice varieties with the ideal plant type.

## Introduction

Leaf is the major organ responsible for photosynthesis and transpiration. In rice (*Oryza sativa* L.), leaf shape is one of the main factors affecting the rice plant type. Thus, it is considered an important agronomic trait determining photosynthetic potential and grain yield in rice (Smith and Dilday, 2002; Yang and Hwa, 2008). The V-shape or moderate rolling of leaves increases canopy photosynthesis by enhancing CO_2_ penetration (Chakraborty, 2001) and improves light capture by reducing mutual shading (Defeng et al., 2001). Therefore, moderately rolled leaves are thought to support higher grain yield than flat leaves (Setter et al., 1995). Moreover, under stress conditions such as high light intensity and excessive transpiration, leaf rolling acts as a stress avoidance mechanism by reducing the leaf surface area (Kadioglu and Terzi, 2007). Therefore, moderate leaf rolling is considered a key trait of high-yielding varieties with the ideal plant type, and this trait has been widely used in the breeding of super hybrid rice cultivars (Denning and Mew, 1997).

Leaf morphology is regulated by a range of cellular processes, including cell development, differentiation, specification, and axis determination (Itoh et al., 2005; Zou et al., 2014). The main factors determining leaf morphology include bulliform cells, sclerenchyma cells, adaxial–abaxial axis polarity, and cuticle development (Xu et al., 2018). To date, several genes have been isolated that regulate leaf rolling by altering these histological characteristics of leaf. *Semi rolled leaf 1 (SRL1)* regulates leaf rolling by inhibiting the formation of bulliform cells. Rice *srl1* mutants show an increased number of bulliform cells on the adaxial surface of the leaf blade, causing adaxial rolling (Xiang et al., 2012). By contrast, mutation of the *narrow leaf 7 (NAL7)* gene decreases the size but increases the number of bulliform cells, leading to adaxial leaf rolling (Fujino et al., 2008). *Semi rolled leaf 2 (SRL2)* is involved in the regulation of cell differentiation on the abaxial side (Liu et al., 2016). The *srl2* mutants exhibit abnormal development of sclerenchyma cells on the abaxial surface of leaves, leading to adaxial leaf rolling. *Adaxialized leaf 1 (ADL1)* plays an important role in leaf pattern formation (Hibara et al., 2009). *adl1* mutants exhibit abaxial leaf rolling due to defects in epidermal development, resulting in abnormal adaxial–abaxial polarity. *OsMYB103L* influences the cellulose content and mechanical strength of leaves (Yang et al., 2014) and its overexpression increases the cellulose content of leaves, resulting in adaxially rolled leaves.

Despite the identification of numerous genes controlling leaf morphology, not all genes are suitable for improving the plant type *via* ideotype breeding since some of these genes cause extreme leaf rolling or are associated with unfavorable effects, such as growth retardation, abnormal organ development, and reduced grain yield (Fujino et al., 2008; Hu et al., 2010; Yang et al., 2014; Zhang et al., 2015; Liu et al., 2016; Li et al., 2017). Recently, quantitative trait loci (QTLs) and their alleles promoting moderate leaf rolling have been identified in natural populations of rice using joint linkage-association mapping (Zhang et al., 2016). Six QTLs were simultaneously identified by genome-wide association study (GWAS) and linkage mapping. These QTLs could explain 3.3–12.9% of the variation in the leaf rolling index (LRI) of 262 recombinant inbred lines (RILs), and the favorable alleles of these QTLs increased the LRI of 1,129 rice accessions by 1.4–2.4%. However, the application of these QTLs for improving the leaf rolling phenotype of rice *via* breeding remains unknown. To improve the plant type by controlling leaf rolling *via* molecular breeding, it is necessary to verify that the genetic effects of these QTLs or their favorable alleles will be expressed consistently in recipient plants, and the simultaneous use of multiple QTLs will allow the precise modulation of the leaf rolling phenotype within a wide range.

Milyang 23 (M23), a Korean high-yielding variety, was developed by performing an inter-subspecific cross between *indica* and *japonica*. Since M23 exhibits some desirable traits contributing to high yield, such as erect leaves, short culm length, large number of spikelets per panicle, and increased translocation of non-structural carbohydrate to sink tissues (Nuruzzaman et al., 2000; Kobayashi et al., 2003; Takai et al., 2005), it has been widely used to develop a high-yielding variety as an elite donor (Kaneda, 1986; Lamo et al., 2015). In particular, the erect plant type of M23, which is attributed to its long-wide, straight, and adaxially rolled leaves, contributes to a high canopy photosynthetic rate and high biomass production (Kobayashi et al., 2003; San et al., 2018).

The objectives of this study are (1) to identify the QTLs affecting the adaxial leaf rolling of M23 using a RIL population, (2) to validate the genetic effect of stable QTLs on the degree of leaf rolling in backcross progenies, and (3) to reveal the morphological basis of adaxial leaf rolling by examining the histological characteristics of leaves altered by these QTLs.

## Materials and Methods

### Plant Materials for QTL Mapping and Field Testing Conditions

A total of 162 F_15_ RILs derived from an inter-subspecific cross between Tong 88-7 (T887; *japonica* rice; flat leaf) and Milyang 23 (M23; *indica* rice; moderate rolled leaf) were used for genotyping-by-sequencing (GBS) and QTL analysis. All plant materials were cultivated in the experimental field of Seoul National University, Suwon, South Korea. Field tests (FTs) were conducted during May–October, 2015 (field test 1; FT 1) and 2016 (field test 2; FT 2). Detailed information of the environmental conditions is shown in Supplementary Table 1. Thirty-day-old seedlings were transplanted into the paddy field as follows: one line per row, 25 plants per row, one plant per hill, 15 cm spacing between two plants in a row, and 30 cm spacing between adjacent rows.

### Adaxial Leaf Rolling Evaluation

Adaxial leaf rolling was assessed based on the LRI, which was calculated by the following equation (Shi et al., 2007):

$$\begin{array}{cc} & \text{LRI}\left(\%\right)=(\mathrm{Width}\mathrm{of}\mathrm{fully}\mathrm{expanded}\mathrm{leaf}\mathrm{blade}-\\ & \mathrm{Natural}\mathrm{distance}\mathrm{of}\mathrm{leaf}\mathrm{blade}\mathrm{margin})/\\ & \mathrm{Width}\mathrm{of}\mathrm{fully}\mathrm{expanded}\mathrm{leaf}\mathrm{blade}\times 100. \end{array}$$

us, a higher LRI means more leaf rolling and vice versa. LRI was measured from the center of the flag leaf (FL) and upper secondary leaf (SL) for individual plants, after the neck of the panicle had completely emerged from the leaf sheath. The uppermost stem was not used for measurements because of large variation in leaf size. To avoid the effect of changes in soil water content on leaf rolling, the rice plants were constantly irrigated until the measurements were complete. All statistical analyses of phenotypic values were performed using R studio v1.2.5033 (Allaire, 2012).

### Histological Assay

A 2-cm section of the SL was sampled at the center of the leaf blade and immediately fixed in formalin–acetic acid–alcohol (FAA) solution (10% formalin, 50% ethanol, 5% acetic acid, and 35% water). All samples were stored at 4°C until needed for histological analysis. Transverse sections of the leaf blade were prepared manually using a razor blade. The leaf sections were cleaned several times with distilled water and then mounted on a microscope slide with a drop of water. Histological characteristics of each leaf were observed under the CX31 light microscope (Olympus), and images were captured using an eXcope T500 microscope camera (DIXI Science). The ImageJ software (version 1.51; Schneider et al., 2012) was used to measure various histological characteristics from the images. Large vein (LV) number, small vein (SV) number, and SV:LV ratio was determined in the entire leaf. Leaf thickness parameters were measured across the SVs and bulliform cells (BCs). Leaf thickness across the SV (LTSV) was defined as the distance from the most protruding region of the epidermis on the adaxial surface to the most recessed region of the epidermis on the abaxial surface (Figure 5A). Leaf thickness across the BC (LTBC) was defined as the distance from the most recessed point of the BC on the adaxial surface to the most protruding region of the epidermis on the abaxial surface. BC height was defined as length of the longest longitudinal axis within the BC. Values of these characteristics were used to calculate the ratio of LTBC:LTSV and the proportion of BCs (as shown below):

BCproportion(%)=(BCheight/LTBC)×100.

### GBS and QTL Analysis

Genomic DNA was extracted from young leaves of each RIL using the cetyl trimethylammonium bromide (CTAB) method (Murray and Thompson, 1980), and the quality of the isolated DNA was verified using PicoGreen (Invitrogen). GBS libraries of RILs were constructed as described previously (Elshire et al., 2011). Briefly, DNA (10 ng/μl) was digested with *Ape*KI and ligated to adapters on both ends. The quantity and quality of GBS libraries were determined using the Bioanalyzer Kit (Agilent Genomics), and the libraries were sequenced on the HiSeq 2000 platform. The reference genome sequence was *in silico*-searched for *Ape*KI restriction sites and split into fragments. A subset of the Nipponbare reference genome IRGSP 1.0 (Sakai et al., 2013) with *Ape*KI restriction was prepared for variant calling. A subset of genome fragments smaller than 2-kb were collected and indexed, and the GBS reads were mapped using the Burrows–Wheeler alignment (BWA) tool (Li and Durbin, 2009). The genotype of each RIL was determined using SAMtools, with default parameters (Li et al., 2009). Raw GBS data of 155 out of 162 RILs were deposited in the National Center for Biotechnology Information (NCBI) Short Read Archive (SRA) under the bio project number PRJNA601019 in previous study (Jang et al., 2020). A total of 6,141 markers (missing rate < 30%) were used for the construction of a genetic map, and QTL analysis was performed using ICIMapping 4.1 (Meng et al., 2015). The recombination distance was calculated using the Kosambi mapping function (Kosambi, 1943). The flag leaf rolling index (FLRI) and secondary leaf rolling index (SLRI) data collected from two field tests, FT 1 and FT 2, were analyzed by inclusive composite interval mapping (ICIM) to detect significant QTLs with a logarithm of odds (LOD) score > 2.5.

### Marker Development

To develop molecular markers, polymorphisms between the two parental accessions, T887 and M23, were identified by comparing their whole genome sequence (WGS) data generated on the Illumina HiSeq platform. The WGS data of T887 and M23 were deposited in the NCBI SRA database under the bio sample numbers SAMN13840663 and SAMN03120572, respectively. All gel-based insertion–deletion (InDel) and single-nucleotide polymorphism (SNP) markers were developed using Primer3Plus (Untergasser et al., 2007) and BatchPrimer3 (You et al., 2008; Supplementary Table 2). Each PCR reaction contained more than 50 ng of template DNA, 1 μl of dNTPs (10 mM), 0.5 U of Prime *Taq* polymerase (GeNet Bio), 1 μl of 10 × PCR buffer, and 1 μl of each primer (10 μM). PCR amplification was performed using the following conditions: initial denaturation at 95°C for 10 min; 30 cycles of 95°C for 30 s, 58°C for 30 s, and 72°C for 30 s; and final extension at 72°C for 10 min.

### Validation and Fine Mapping of QTLs

To examine the gene action of two QTLs, *qARO1* and *qARO9*, a BC_2_F_2_ population was developed by backcrossing T887 (donor parent) with M23 (recurrent parent). Backcross progenies harboring the *qARO5* allele of M23 were selected using three markers. *qARO1* and *qARO9* alleles of T887 were identified using seven and five markers, respectively (Supplementary Table 2). Near-isogenic lines (NILs) carrying both *qARO1* and *qARO9* or only *qARO9* was selected from each individual BC_3_F_2_ plant with the genetic background recovered to the recurrent parent. Marker-assisted background selection for developing NILs was conducted using 186 markers, including 178 SNPs (Fluidigm platform), which evenly cover the rice genome (Seo et al., 2020), and eight InDel markers (Supplementary Table 2). The BC_3_F_3_ population was employed for fine mapping of *qARO1* and *qARO9* using 1,860 and 368 individuals, respectively.

## Results

### Variation in the Leaf Rolling Phenotype of RILs

**FIGURE 1:**
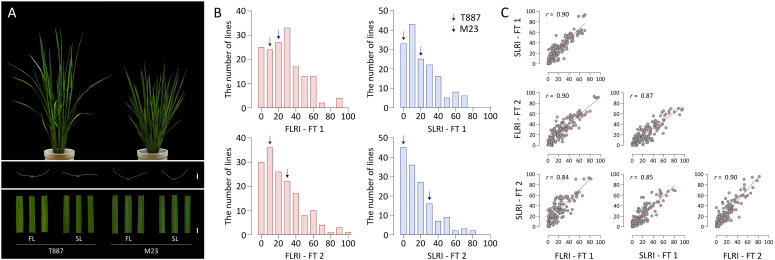
Analysis of the leaf rolling index (LRI) of the flag leaf (FL) and secondary leaf (SL) of recombinant inbred lines (RILs). **(A)** Plant type and leaf shape of both parents of the RIL population. From top to bottom: plant type at the vegetative stage; transverse sections of the FL and SL (scale bar = 2 mm); shapes of FL and SL (scale bar = 10 mm). **(B)** Variation in the LRI of the FL (FLRI) and SL (SLRI) of RILs in field test 1 (FT 1) and field test 2 (FT 2). Red and blue arrows indicate average phenotypic values of the parents, Tong 88-7 (T887) and Milyang 23 (M23), respectively. **(C)** Correlation between FLRI and SLRI in both field tests. Pearson’s correlation coefficients (*r*) were employed to estimate strength of correlation between each phenotype.

The high-yielding Korean rice variety M23 showed a straighter plant type, with erect leaves, than T887. Leaves of M23 showed moderate rolling, whereas those of T887 were relatively flat (Figure 1A). In the two FTs, the FLRI and SLRI of T887 ranged from 10.3 to 14.1% and 3.1 to 5.4%, respectively, whereas those of M23 ranged from 23.8 to 24.1% and 26.5 to 29.3%, respectively (Figure 1B). Among the RILs derived from a cross between T887 and M23, the value of FLRI varied from 0% (fully flat leaf) to 95.8% (extremely rolled leaf) in the two FTs. Similarly, the SLRI of RILs varied from 0 to 81.8%. A number of RILs showed higher SLRI and FLRI values than both parents, indicating transgressive segregation (Figure 1B and Supplementary Table 3). A strong positive correlation (*r* = 0.9) was detected between the FLRI and SLRI values of RILs each year, as well as between the two FTs for FLRI (*r* = 0.9) and SLRI (*r* = 0.85) (Figure 1C).

### QTL Analysis of the LRI of RILs

**FIGURE 2:**
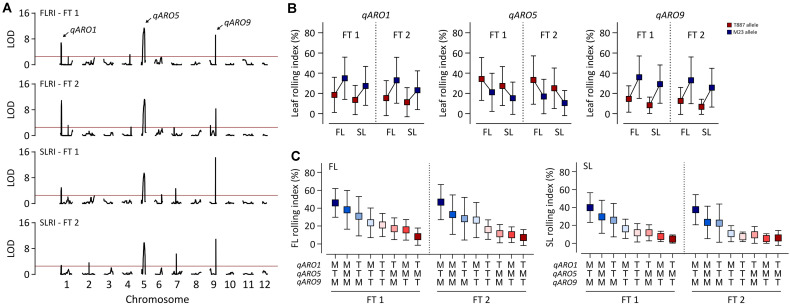
Detection of quantitative trait loci (QTLs) controlling adaxial leaf rolling in rice using the RIL population. **(A)** QTL mapping for FLRI and SLRI. The horizontal red line denotes the threshold LOD score for detecting significant QTLs (LOD > 2.5). Black curve indicates the LOD score of FLRI and SLRI. Arrows above the LOD curve indicate the stable QTLs controlling adaxial leaf rolling in both tests. **(B)** Variation in LRI among RILs, depending on the allele type at the three stable QTLs, *qARO1*, *qARO5*, and *qARO9*. **(C)** Effects of the allelic combination of *qARO1*, *qARO5*, and *qARO9* on FLRI and SLRI.

A total of 6,140 markers were mapped onto the entire Nipponbare genome, covering 1,278.1 cM in 162 RILs, with the following genomic distribution: on average, 511.7 markers per chromosome, 4.8 markers per cM (genetic map), and 16.5 markers per Mb (physical map) (Supplementary Table 4). A total of 12 and 10 significant QTLs were detected for FLRI and SLRI, respectively, in both FTs (Figure 2A and Supplementary Table 5). Among these, three QTLs were simultaneously detected for FLRI and SLRI in the two FTs. These three stable QTLs conferring adaxial leaf rolling were named *adaxially rolled leaf 1* (*qARO1*), *qARO5*, and *qARO9*; these three QTLs were detected on the short arm of chromosome 1 (4.05–4.67 Mb), the long arm of chromosome 5 (20.41–23.05 Mb), and the long arm of chromosome 9 (19.29–19.7 Mb), respectively (Figure 2A and Supplementary Table 5). The phenotypic variance explained (PVE) values of *qARO1* for FLRI (10–15.2%) and SLRI (4.5–7%) were lower than those of *qARO5* (FLRI: 18.3–21%; SLRI: 16.5–16.7%) and *qARO9* (FLRI: 11.3–14.1%; SLRI: 18.2–23.9%) (Figure 2A and Supplementary Table 5). Among these three QTLs, *qARO1* and *qARO9* showed positive additive effects, implying that M23 alleles at both these QTLs (*qARO1*^M23^ and *qARO9*^M23^, respectively) contribute to the leaf rolling phenotype. By contrast, *qARO5* showed a negative additive effect, and the T887 allele at *qARO5* (*qARO5*^T887^) increased the LRI (Figure 2B and Supplementary Table 5). RILs containing a combination of *qARO1*^M23^, *qARO5*^T887^, and *qARO9*^M23^ alleles showed the highest average FLRI and SLRI values in both FTs, whereas those harboring the *qARO1*^T887^, *qARO5*^M23^, and *qARO9*^T887^ alleles showed the lowest FLRI and SLRI values, except for SLRI in FT 2 (Figure 2C).

### Gene Action of *qARO1* and *qARO9* in the BC_2_F_2_ Population

**FIGURE 3:**
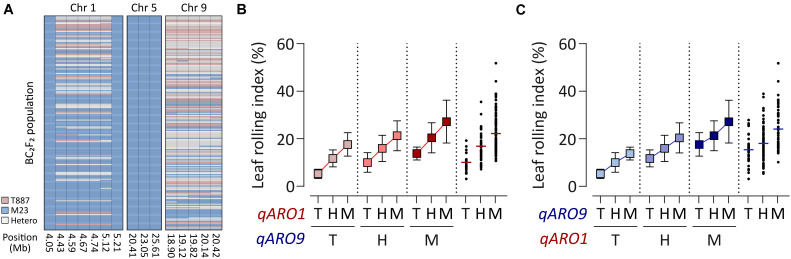
Gene action of *qARO1* and *qARO9* in the BC_2_F_2_ population. **(A)** Genotypes of *qARO1*, *qARO5*, and *qARO9*. Pink, blue, and gray colors indicate T887, M23, and heterozygous alleles, respectively. **(B,C)** Allelic effect of *qARO1* **(B)** or *qARO9* **(C)**, in combination with the other QTL on LRI. T, T887 allele; M, M23 allele; H, heterozygous allele.

Since RILs do not contain heterozygous alleles of QTLs, the gene action of *qARO1* and *qARO9* was examined in the BC_2_F_2_ population, which was developed using M23 as the recurrent parent. Backcross progenies of plants harboring the donor allele at *qARO5* (*qARO5*^M23^) and heterozygous alleles at *qARO1* and *qARO9* were selected (Figure 3A). The *qARO1*^M23^ and *qARO9*^M23^ alleles conferred higher LRI in the BC_2_F_2_ population, consistent with the results of primary mapping (Figure 2A). The LRI values of plants harboring the heterozygous alleles at *qARO1* and *qARO9* were intermediate between the two parental LRI values, indicating that *qARO1* and *qARO9* exhibit additive gene actions (Figures 3B,C). Furthermore, both QTLs showed additive gene action, regardless of the allele type of the other QTL, implying the absence of epistatic interactions between two QTLs (Figures 3B,C).

### Validation of the Effects of *qARO1* and *qARO9* Using NILs

**FIGURE 4:**
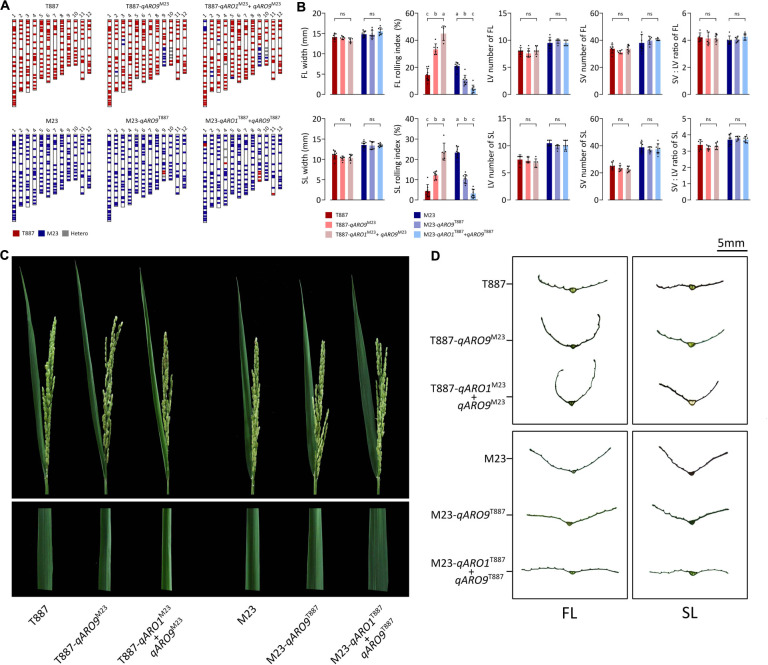
Near-isogenic lines (NILs) possessing *qARO1* and *qARO9*. **(A)** Background genotypes of NILs backcrossed to both parental directions. **(B)** Comparison of FL- and SL-related traits among NILs and parents. Different lowercase letters indicate statistically significant differences [one-way analysis of variance (ANOVA), followed by Scheffe’s *post hoc* test; *p* < 0.05]. ns, non-significant. **(C)** Adaxial rolling of the FL of NILs and both parents after the completion of panicle emergence. **(D)** Transverse sections of the FL and SL.

Each NIL was developed by backcrossing with both parents and then was genotyped for *qARO1* (markers: 01id4052916 and 01id5210929) and *qARO9* (markers: 09id18904919 and 09id19950022). Each NIL was selected from the individual plant with the genetic background recovered maximally to the recipient parent, carrying *qARO1* and/or *qARO9* alleles from the donor parent. Consequently, four NILs including T887-*qARO9*^M23^, T887-*qARO1*^M23^+*ARO9*^M23^, M23-*qARO9*^T887^, and M23-*qARO1*^T887^+*qARO9*^T887^ were successfully developed. Among these, T887-*qARO9*^M23^ and T887-*qARO1*^M23^+*ARO9*^M23^ recovered 91.4% (170 out of 186 markers) and 87.6% (163 out of 186 markers) of the T887 genetic background, while M23-*qARO9*^T887^ and M23-*qARO1*^T887^+*qARO9*^T887^ recovered 97.3% (181 out of 186 markers) and 93.6% (174 out of 186 markers) of the M23 background (Figure 4A). Both *qARO1* and *qARO9* showed additive effects on FLRI and SLRI, regardless of the genetic background. Significant differences were detected in FLRI and SLRI among NILs and their parent, but no significant changes were observed in leaf width, panicle number, panicle dry weight, and plant dry weight (Figures 4B–D and Supplementary Table 6).

### Histological Analysis of NILs

**FIGURE 5:**
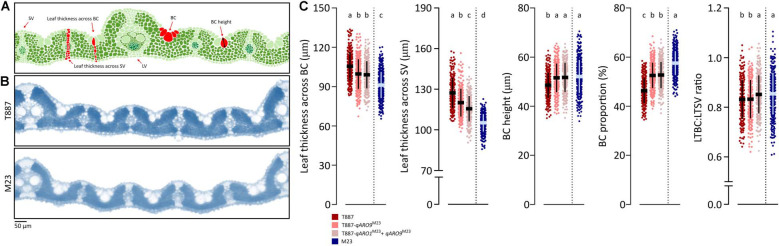
Histological analysis of NILs and their parents. **(A)** Measurements of histological traits. LV, large vein; SV, small vein; BC, bulliform cell. **(B)** Transverse leaf sections of both parents. **(C)** Comparison of histological characteristics among NILs (T887 genetic background) and both parents (one-way ANOVA, followed by Scheffe’s *post hoc* test; *p* < 0.05). The horizontal and vertical lines on the dot plot indicate average values and standard deviation, respectively.

To determine the effects of *qARO1* and *qARO9* on adaxial leaf rolling, histological assays were conducted (Figure 5A). The LV number, SV number, and SV:LV ratio in both FL and SL showed no significant differences between NILs and their parents, suggesting that vein number is not involved in the regulation of the leaf rolling phenotype (Figure 4B). More precisely, five characteristics including LTBC, LTSV, LTBC:LTSV ratio, BC height, and BC proportion were compared among NILs with the T887 genetic background and both parents (Figures 5B,C). Among these five characteristics, LTBC, BC height, and BC proportion showed significant differences between T887 and two NILs but not between T887-*qARO9*^M23^ and T887-*qARO1*^M23^+*ARO9*^M23^. LTSV was significantly different not only between T887 and two NILs but also between T887-*qARO9*^M23^ and T887-*qARO1*^M23^+*ARO9*^M23^. The LTBC:LTSV ratio showed significant differences between T887-*qARO9*^M23^ and T887-*qARO1*^M23^+*ARO9*^M23^ but not between T887 and T887-*qARO9*^M23^ (Figure 5C).

### Fine Mapping of qARO1 and qARO9 Using the BC_3_F_3_ Population

**FIGURE 6:**
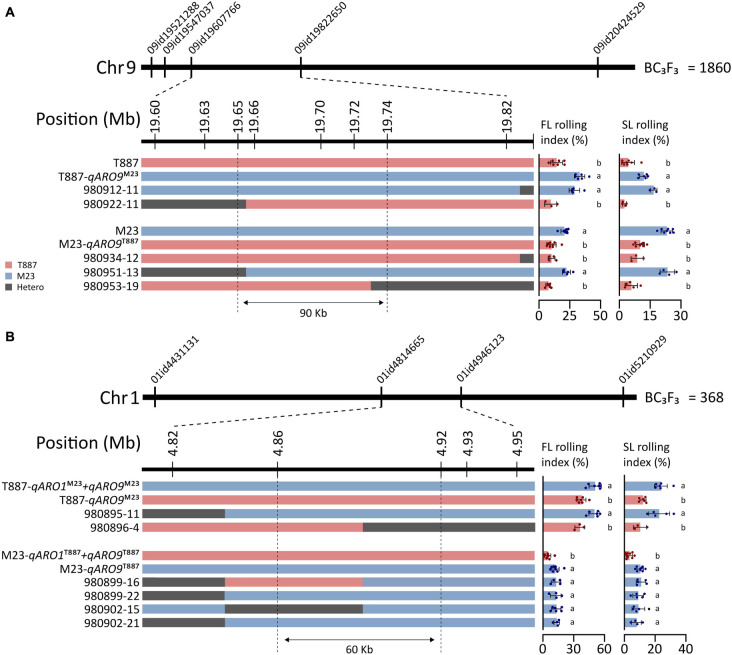
Fine mapping of *qARO1* and *qARO9* using the BC_3_F_3_ population. **(A,B)** Delimitation of *qARO9* **(A)** and *qARO1* **(B)**. Red, blue, and gray colors denote T887, M23, and heterozygous genotype. Values of FLRI and SLRI were compared using one-way ANOVA, followed by Scheffe’s *post hoc* test (*p* < 0.05).

To further narrow down the region of chromosomes 1 and 9 where *qARO1* and *qARO9* are located, we developed a BC_3_F_3_ population comprising 2,228 plants by backcrossing in both parental directions, T887 (924 plants) and M23 (1,304 plants). The *qARO9* region was delimited to an interval of 0.22-Mb on chromosome 9 (19.6–19.82 Mb) between marker 09id19607766 and marker 09id19822650 using 1,860 plants derived from the ancestor plants harboring the recipient allele of *qARO1* and *qARO5*. Six additional markers were used to narrow down the target region in five recombinant plants. Multiple comparisons revealed that recombinants were classified into two groups, recurrent parent and NIL-*qARO9*, based on the average FLRI and SLRI values. Thus, *qARO9* was delimited to a 90-kb interval (19.65–19.74 Mb) (Figure 6A).

*qARO1* was fine-mapped using the 368 plants derived from the ancestor plants with the recipient allele of *qARO5* and donor allele of *qARO9* to clearly discriminate the *qARO1* effect on LRI. Consequently, *qARO1* was mapped to a 0.13-Mb interval (4.82–4.95 Mb) on chromosome 1 between markers 01id4814665 and 01id4946123 using the entire population. A total of six recombinants were identified within this interval. Using three additional markers, *qARO1* was delimited to a 60-kb interval (4.86–4.92 Mb) (Figure 6B).

## Discussion

Moderate leaf rolling is considered a desired leaf trait for high yielding varieties of rice with the ideal plant type (Setter et al., 1995; Denning and Mew, 1997; Chakraborty, 2001; Defeng et al., 2001). Therefore, identification of loci that modulate leaf rolling and dissection of the genetic effects of these loci could facilitate the development of cultivars with the ideal plant type. In this study, we performed QTL mapping to detect stable QTLs that regulate adaxial leaf rolling in rice using a RIL population. Additionally, to validate the genetic effects of QTLs on adaxial leaf rolling, we investigated the morphological and histological characteristics of leaves using backcross progenies.

In the RIL population derived from a cross between T887 and M23, the FLRI and SLRI values showed transgressive segregation in the positive direction, implying that the genetic factors responsible for adaxial leaf rolling could have originated from either parent (Figure 1B). Among the three stable QTLs that simultaneously affected FLRI and SLRI in both FTs, *qARO1* and *qARO9* showed positive additive effects on LRI, while *qARO5* showed a negative additive effect, suggesting that alleles causing adaxial leaf rolling were derived from each parent belonging to a different subspecies of rice, *japonica* and *indica*. A similar result was reported previously (Zhang et al., 2016). The authors reported highly transgressive variation in the LRI trait in the RIL population derived from a cross between Minghui 63 (*indica* rice) and 02428 (*japonica* rice). Moreover, among six stable QTLs affecting LRI, the positive alleles of four loci were derived from 02428, while positive alleles of the other QTLs were derived from Minghui 63. Taken together, these results suggest that alleles causing adaxial leaf rolling might be distributed in different subspecies and that introducing allelic combinations of these QTLs by inter-subspecific crossing is an efficient strategy for modulating the degree of leaf rolling within a diverse range.

Several previous studies have reported several QTLs for leaf rolling on chromosomes 1, 5, and 9. Based on the IRGSP 1.0 database (Sakai et al., 2013), *qRl5-4* is located at the 20.51- to 21.99-Mb position on chromosome 5 and *qRl9-1* is located at the 7.96- to 10.90-Mb position on chromosome 9 (Zhang et al., 2016). In addition, *qRL-1*, represented by the nearest marker RM3453, is located near the 4.89-Mb position on chromosome 1 and *qRL-9*, represented by the nearest marker RM8206, is located near the 5.92-Mb position on chromosome 9 (Guo et al., 2010). Among these QTLs, *qRL-1* and *qRl5-4* were co-located with *qARO1* (4.86–4.92 Mb) and *qARO5* (20.41–23.05 Mb), respectively (Figure 6B and Supplementary Table 5), while *qARO9* was newly detected in this study.

A relatively wide range of FLRI (3.3–45.9%) and SLRI (4.6–39.9%) was observed in the RIL population, depending on the allelic combination of *qARO1*, *qARO5*, and *qARO9*. These three QTLs explained on average 45 and 43.4% of the phenotypic variance in FLRI and SLRI, respectively (Figure 2D and Supplementary Table 5). In addition, accumulation of the genetic effects of *qARO1* and *qARO9* in NILs changed the leaf shape from almost flat to moderately rolled (Figure 4B). These results suggest that *qARO1*, *qARO5*, and *qARO9* precisely regulate the leaf rolling phenotype within a wide range and therefore are ideal candidates for application in molecular breeding programs.

Although several genes controlling the leaf rolling phenotype have been cloned *via* genetic mapping of mutant plants (Fujino et al., 2008; Hibara et al., 2009; Xiang et al., 2012; Yang et al., 2014; Liu et al., 2016), these mutant genes exhibit several limitations that prohibit their use in breeding programs. For example, mutant genes cause severe leaf rolling, which leads to developmental defects and grain yield reduction (Li et al., 2017). Moreover, mutant genes often cause unfavorable effects on other agronomic traits. Almost all leaf rolling mutants show recessive inheritance; thus, both male and female parents must possess the same recessive gene to modulate leaf rolling in super hybrid rice, the development of which is time-consuming and labor-intensive (Wu et al., 2018). Since genotypes heterozygous at *qARO1* and *qARO9* showed intermediate values of LRI compared with the parents (Figure 3A), and regulated adaxial leaf rolling without significant defects in leaf width, panicle biomass, or plant biomass of individual plants (Figure 4B and Supplementary Table 6), these QTLs could be used to improve the leaf shape of rice cultivars *via* ideotype breeding. However, further investigation is needed to verify whether moderate leaf rolling resulting from the QTLs could increase grain yield at the population level.

Leaf rolling is mainly caused by histological alterations in leaf cells (Xu et al., 2018). Both *qARO1* and *qARO9* regulated adaxial leaf rolling by affecting different histological characteristics of leaves. The T887-*qARO9*^M23^ NIL showed significantly lower leaf thickness parameters (LTBC and LTSV) but higher BC height and BC proportion than T887 (Figure 5C). The T887-*qARO1*^M23^+*qARO9*^M23^ NIL showed further reduction in LTSV, resulting in higher LTBC:LTSV ratio and greater leaf rolling compared with T887-*qARO9*^M23^ (Figure 5C). Overall, our data suggest that *qARO9* is involved in the determination of thickness in the entire leaf, as well as BC size, whereas *qARO1* is involved in the determination of the ratio of leaf thickness by regulating leaf thickness near the SV. This implies that *qARO1*- and *qARO9*-mediated reduction in leaf thickness in specific regions of the leaf and an increase in the proportion of BC alter the leaf structure, resulting in adaxial leaf rolling.

## Data Availability Statement

The datasets presented in this study can be found in online repositories. The names of the repository/repositories and accession number(s) can be found in the article/Supplementary Material.

## Author Contributions

SJ designed the experiment and prepared the manuscript. SJ and DL conducted the field experiments. SJ and YL conducted the wet-laboratory experiments. SS conducted computational analysis. H-JK supervised the work and contributed to the finalization of the manuscript. All authors read and approved the manuscript.

## Conflict of Interest

The authors declare that the research was conducted in the absence of any commercial or financial relationships that could be construed as a potential conflict of interest.

## References

[B1] Allaire J. (2012). *RStudio: Integrated Development Environment for R.* Vol. 537. Boston, MA: RStudio, 538.

[B2] Chakraborty S. (2001). *Rice Breeding and Genetics.* Delhi: Concept Publishing Company.

[B3] Defeng Z. Xianqing L. Weixing C. (2001). Comparison of leaf photosynthetic characteristics among rice hybrids with different leaf rolling index. *Zuo Wu Xue Bao* 27 329–333.

[B4] Denning G. L. Mew T. W. (1997). *“China and IRRI” Improving China’s Rice Productivity in the 21st Century.* Los Baños: International Rice Research Institute.

[B5] Elshire R. J. Glaubitz J. C. Sun Q. Poland J. A. Kawamoto K. Buckler E. S. (2011). A robust, simple genotyping-by-sequencing (GBS) approach for high diversity species. *PLoS One* 6:e19379. 10.1371/journal.pone.0019379 21573248 PMC3087801

[B6] Fujino K. Matsuda Y. Ozawa K. Nishimura T. Koshiba T. Fraaije M. W. (2008). NARROW LEAF 7 controls leaf shape mediated by auxin in rice. *Mol. Genet. Genomics* 279 499–507. 10.1007/s00438-008-0328-3 18293011

[B7] Guo Y. Cheng B. Hong D. (2010). Construction of SSR linkage map and analysis of QTLs for rolled leaf in japonica rice. *Rice Sci.* 17 28–34. 10.1016/S1672-6308(08)60101-8

[B8] Hibara K. Obara M. Hayashida E. Abe M. Ishimaru T. Satoh H. (2009). The ADAXIALIZED LEAF1 gene functions in leaf and embryonic pattern formation in rice. *Dev. Biol.* 334 345–354. 10.1016/j.ydbio.2009.07.042 19665012

[B9] Hu J. Zhu L. Zeng D. Gao Z. Guo L. Fang Y. (2010). Identification and characterization of NARROW AND ROLLED LEAF 1, a novel gene regulating leaf morphology and plant architecture in rice. *Plant Mol. Biol.* 73 283–292. 10.1007/s11103-010-9614-7 20155303

[B10] Itoh J.-I. Nonomura K.-I. Ikeda K. Yamaki S. Inukai Y. Yamagishi H. (2005). Rice plant development: from zygote to spikelet. *Plant Cell Physiol.* 46 23–47. 10.1093/pcp/pci501 15659435

[B11] Jang S. Han J.-H. Lee Y. K. Shin N.-H. Kang Y. J. Kim C.-K. (2020). Mapping and validation of QTLs for the amino acid and total protein content in brown rice. *Front. Genet.* 11:240. 10.3389/fgene.2020.00240 32256527 PMC7089939

[B12] Kadioglu A. Terzi R. (2007). A dehydration avoidance mechanism: leaf rolling. *Bot. Rev.* 73 290–302. 10.1663/0006-8101(2007)73[290:adamlr]2.0.co;2

[B13] Kaneda C. (1986). Rice breeding for extremely higher yielding ability by japonica-indica hybridization. *Japan Agric. Res. Q.* 19 235–240.

[B14] Kobayashi S. Fukuta Y. Morita S. Sato T. Osaki M. Khush G. S. (2003). Quantitative trait loci affecting flag leaf development in rice (*Oryza sativa* L.). *Breed. Sci.* 53 255–262. 10.1270/jsbbs.53.255 26081539

[B15] Kosambi D. D. (1943). The estimation of map distances from recombination values. *Ann. Eugen.* 12 172–175. 10.1111/j.1469-1809.1943.tb02321.x

[B16] Lamo J. Cho G. Jane I. Dartey P. K. A. James E. Ekobu M. (2015). Developing lowland rice germplasm with resistance to multiple biotic stresses through anther culture in Uganda. *Korean J. Int. Agric.* 27 415–420. 10.12719/KSIA.2015.27.4.415

[B17] Li H. Durbin R. (2009). Fast and accurate short read alignment with Burrows–Wheeler transform. *Bioinformatics* 25 1754–1760. 10.1093/bioinformatics/btp324 19451168 PMC2705234

[B18] Li H. Handsaker B. Wysoker A. Fennell T. Ruan J. Homer N. (2009). The sequence alignment/map format and SAMtools. *Bioinformatics* 25 2078–2079. 10.1093/bioinformatics/btp352 19505943 PMC2723002

[B19] Li W.-Q. Zhang M.-J. Gan P.-F. Qiao L. Yang S.-Q. Miao H. (2017). *CLD1* / *SRL1* modulates leaf rolling by affecting cell wall formation, epidermis integrity and water homeostasis in rice. *Plant J.* 92 904–923. 10.1111/tpj.13728 28960566

[B20] Liu X. Li M. Liu K. Tang D. Sun M. Li Y. (2016). *Semi-Rolled Leaf2* modulates rice leaf rolling by regulating abaxial side cell differentiation. *J. Exp. Bot.* 67 2139–2150. 10.1093/jxb/erw029 26873975 PMC4809286

[B21] Meng L. Li H. Zhang L. Wang J. (2015). QTL IciMapping: integrated software for genetic linkage map construction and quantitative trait locus mapping in biparental populations. *Crop J.* 3 269–283. 10.1016/j.cj.2015.01.001

[B22] Murray M. G. Thompson W. F. (1980). Rapid isolation of high molecular weight plant DNA. *Nucleic Acids Res.* 8 4321–4326. 10.1093/nar/8.19.4321 7433111 PMC324241

[B23] Nuruzzaman M. Yamamoto Y. Yoshida T. Nitta Y. Miyazaki A. (2000). Characterization of indica and japonica rice varieties based on improved plant type index. *Jpn. J. Trop. Agric.* 44 77–86.

[B24] Sakai H. Lee S. S. Tanaka T. Numa H. Kim J. Kawahara Y. (2013). Rice annotation project database (RAP-DB): an integrative and interactive database for rice genomics. *Plant Cell Physiol.* 54:e6. 10.1093/pcp/pcs183 23299411 PMC3583025

[B25] San N. S. Yamashita M. Adachi S. Tanabata T. Ookawa T. Hirasawa T. (2018). Differences in lamina joint anatomy cause cultivar differences in leaf inclination angle of rice. *Plant Prod. Sci.* 21 302–310. 10.1080/1343943X.2018.1500488

[B26] Schneider C. A. Rasband W. S. Eliceiri K. W. (2012). NIH Image to ImageJ: 25 years of image analysis. *Nat. Methods* 9 671–675. 10.1038/nmeth.2089 22930834 PMC5554542

[B27] Seo J. Lee G. Jin Z. Kim B. Chin J. H. Koh H.-J. (2020). Development and application of indica–japonica SNP assays using the Fluidigm platform for rice genetic analysis and molecular breeding. *Mol. Breed.* 40:39. 10.1007/s11032-020-01123-x

[B28] Setter T. Conocono E. Egdane J. Kropff M. (1995). Possibility of increasing yield potential of rice by reducing panicle height in the canopy. I. Effects of panicles on light interception and canopy photosynthesis. *Funct. Plant Biol.* 22 441–451. 10.1071/PP9950441

[B29] Shi Z. Wang J. Wan X. Shen G. Wang X. Zhang J. (2007). Over-expression of rice OsAGO7 gene induces upward curling of the leaf blade that enhanced erect-leaf habit. *Planta* 226 99–108. 10.1007/s00425-006-0472-0 17216479

[B30] Smith C. W. Dilday R. H. (2002). *Rice: Origin, History, Technology, and Production.* Hoboken, NJ: John Wiley & Sons.

[B31] Takai T. Fukuta Y. Shiraiwa T. Horie T. (2005). Time-related mapping of quantitative trait loci controlling grain-filling in rice (*Oryza sativa* L.). *J. Exp. Bot.* 56 2107–2118. 10.1093/jxb/eri209 15983016

[B32] Untergasser A. Nijveen H. Rao X. Bisseling T. Geurts R. Leunissen J. A. (2007). Primer3Plus, an enhanced web interface to Primer3. *Nucleic Acids Res.* 35 W71–W74. 17485472 10.1093/nar/gkm306 PMC1933133

[B33] Wu Z. Tang D. Liu K. Miao C. Zhuo X. Li Y. (2018). Characterization of a new semi-dominant dwarf allele of SLR1 and its potential application in hybrid rice breeding. *J. Exp. Bot.* 69 4703–4713. 10.1093/jxb/ery243 29955878 PMC6137977

[B34] Xiang J.-J. Zhang G.-H. Qian Q. Xue H.-W. (2012). SEMI-ROLLED LEAF1 encodes a putative glycosylphosphatidylinositol-anchored protein and modulates rice leaf rolling by regulating the formation of bulliform cells. *Plant Physiol.* 159 1488–1500. 10.1104/pp.112.199968 22715111 PMC3425193

[B35] Xu P. Ali A. Han B. Wu X. (2018). Current advances in molecular basis and mechanisms regulating leaf morphology in rice. *Front. Plant Sci.* 9:1528. 10.3389/fpls.2018.01528 30405666 PMC6206276

[B36] Yang C. Li D. Liu X. Ji C. Hao L. Zhao X. (2014). OsMYB103L, an R2R3-MYB transcription factor, influences leaf rolling and mechanical strength in rice (*Oryza sativa* L.). *BMC Plant Biol.* 14:158. 10.1186/1471-2229-14-158 24906444 PMC4062502

[B37] Yang X. C. Hwa C. M. (2008). Genetic modification of plant architecture and variety improvement in rice. *Heredity* 101 396–404. 10.1038/hdy.2008.90 18716608

[B38] You F. M. Huo N. Gu Y. Q. Luo M.-C. Ma Y. Hane D. (2008). BatchPrimer3: a high throughput web application for PCR and sequencing primer design. *BMC Bioinformatics* 9:253. 10.1186/1471-2105-9-253 18510760 PMC2438325

[B39] Zhang J.-J. Wu S.-Y. Jiang L. Wang J.-L. Zhang X. Guo X.-P. (2015). A detailed analysis of the leaf rolling mutant *sll2* reveals complex nature in regulation of bulliform cell development in rice (*Oryza sativa* L.). *Plant Biol. J.* 17 437–448. 10.1111/plb.12255 25213398

[B40] Zhang Q. Zheng T. Hoang L. Wang C. Nafisah, Joseph C. (2016). Joint mapping and allele mining of the rolled leaf trait in rice (*Oryza sativa* L.). *PLoS One* 11:e0158246. 10.1371/journal.pone.0158246 27441398 PMC4956317

[B41] Zou L. Zhang Z. Qi D. Peng M. Lu T. (2014). Cytological mechanisms of leaf rolling in rice. *Crop Sci.* 54 198–209. 10.2135/cropsci2013.03.0199

