# Supplementary material for: Major QTLs, qARO1 and qARO9, Additively Regulate Adaxial Leaf Rolling in Rice
Source: Front Plant Sci. 2021 Feb 19;12:626523. doi: 10.3389/fpls.2021.626523 (PMC7940999; doi:10.3389/fpls.2021.626523)
Supplement: Supplementary file 1 [file Data_Sheet_1.docx]

Supplementary Material

**Supplementary Table S1. Environmental conditions.**

| Location | Years | Months | Temperature (°C) | | | Relative  humidity (%) | Precipitation (mm) | Sunshine duration  (h) | Solar radiation (MJ/m^2^) |
| --- | --- | --- | --- | --- | --- | --- | --- | --- | --- |
|  |  |  | Mean | Max. | Min. |  |  |  |  |
| Suwon | 2015 | May | 18.2 | 24.9 | 12.2 | 63.0 | 32.6 | 278.8 | 561.9 |
|  |  | June | 23.1 | 29.0 | 18.2 | 66.0 | 30.2 | 245.3 | 519.1 |
|  |  | July | 25.5 | 30.0 | 22.1 | 78.0 | 225.8 | 165.8 | 412.1 |
|  |  | August | 26.2 | 31.3 | 22.5 | 78.0 | 71.0 | 205.7 | 439.4 |
|  |  | September | 22.1 | 27.9 | 16.9 | 66.0 | 6.9 | 241.6 | 420.0 |
|  | 2016 | May | 19.1 | 25.9 | 13.1 | 65.0 | 156.4 | 272.8 | 548.7 |
|  |  | June | 23.2 | 28.9 | 18.8 | 73.0 | 37.4 | 235.6 | 515.2 |
|  |  | July | 26.0 | 30.2 | 22.9 | 83.0 | 317.7 | 146.8 | 392.9 |
|  |  | August | 27.7 | 32.8 | 24.0 | 73.0 | 73.0 | 230.9 | 465.9 |
|  |  | September | 22.7 | 27.6 | 18.9 | 75.0 | 67.8 | 163.2 | 334.7 |
| Max., maximum temperature; Min, minimum temperature. | | | | | | | | | |

**Supplementary Table S3.** **Variation in leaf rolling index (LRI) among recombinant inbred lines (RILs) and both of their parents (T887 and M23).**

|  | Field test 1 (FT 1)^a^ | | Field test 2 (FT 2)^a^ | |
| --- | --- | --- | --- | --- |
|  | FLRI | SLRI | FLRI | SLRI |
| Number of values | 158 | 158 | 158 | 147 |
| LRI range | 0–93.3 | 0–70.4 | 0–95.8 | 0–81.8 |
| Average LRI | 27.8 | 21.3 | 25.2 | 12.5 |
| Coefficient of variation | 0.76 | 0.88 | 0.88 | 1.02 |
|  |  |  |  |  |
| Average LRI of T887 | 10.3 | 5.4 | 14.1 | 3.1 |
| Average LRI of M23 | 23.8 | 24.1 | 29.3 | 26.5 |

^a^FLRI, flag leaf rolling index; SLRI, secondary leaf rolling index.

**Supplementary Table S4.** **Statistics of the genetic map.**

| Chr. | Marker number | Genetic distance (cM) | Physical map (Mb) | No. of markers per cM | No. of markers per Mb |
| --- | --- | --- | --- | --- | --- |
| 1 | 400 | 148.8 | 43.3 | 2.7 | 9.2 |
| 2 | 1243 | 148.6 | 35.9 | 8.4 | 34.6 |
| 3 | 441 | 110.5 | 36.4 | 4.0 | 12.1 |
| 4 | 561 | 120.8 | 35.5 | 4.6 | 15.8 |
| 5 | 44 | 54.2 | 30.0 | 0.8 | 1.5 |
| 6 | 262 | 93.1 | 31.3 | 2.8 | 8.4 |
| 7 | 1387 | 120.6 | 29.7 | 11.5 | 46.7 |
| 8 | 443 | 117.2 | 28.4 | 3.8 | 15.6 |
| 9 | 104 | 82.7 | 23.0 | 1.3 | 4.5 |
| 10 | 593 | 98.6 | 23.2 | 6.0 | 25.5 |
| 11 | 560 | 123.4 | 29.0 | 4.5 | 19.3 |
| 12 | 102 | 59.6 | 27.5 | 1.7 | 3.7 |
| Total | 6140 | 1278.1 | 373.3 | 4.8 | 16.5 |

**Supplementary Table S5.** **Summary of QTLs affecting FLRI and SLRI.**

| Field test | Trait | Chr.^a^ | QTL position (Mb)^b^ | Left marker^b^ | Right marker^b^ | LOD | PVE (%) | Add |
| --- | --- | --- | --- | --- | --- | --- | --- | --- |
| FT 1 | FLRI | 1 | **4.05 - 4.67** | **01id_4052916** | **01id_4673121** | 6.9 | 10.0 | -6.8 |
|  |  | 1 | 27.56 - 27.57 | sch01_27560281 | sch01_27566411 | 2.6 | 3.7 | -4.0 |
|  |  | 4 | 31.03 - 31.07 | sch04_31030709 | sch04_31070764 | 3.3 | 4.4 | 4.6 |
|  |  | 5 | **20.41 - 23.05** | **05id_20410437** | **05id_23047412** | 11.4 | 21.0 | 9.6 |
|  |  | 9 | **19.29 - 19.7** | **sch09_19288183** | **sch09_19699833** | 9.3 | 14.1 | -8.3 |
|  | SLRI | 1 | **4.05 - 4.67** | **01id_4052916** | **01id_4673121** | 5.1 | 7.0 | -5.0 |
|  |  | 5 | **20.41 - 23.05** | **05id_20410437** | **05id_23047412** | 9.4 | 16.7 | 7.7 |
|  |  | 6 | 23.36 - 23.45 | sch06_23360101 | sch06_23448492 | 2.9 | 3.8 | -3.8 |
|  |  | 7 | 7.78 - 7.91 | sch07_7781158 | sch07_7912666 | 4.7 | 6.5 | -4.8 |
|  |  | 9 | **19.29 - 19.7** | **sch09_19288183** | **sch09_19699833** | 14.4 | 23.9 | -9.6 |
| FT 2 | FLRI | 1 | **4.05 - 4.67** | **01id_4052916** | **01id_4673121** | 11.0 | 15.2 | -8.7 |
|  |  | 1 | 26.42 - 26.7 | sch01_26424385 | sch01_26701198 | 3.3 | 4.1 | -4.5 |
|  |  | 4 | 32.41 - 32.59 | ich04_32414834 | sch04_32591693 | 2.8 | 3.4 | 4.1 |
|  |  | 5 | **20.41 - 23.05** | **05id_20410437** | **05id_23047412** | 11.3 | 18.3 | 9.4 |
|  |  | 7 | 4.19 - 4.22 | sch07_4194621 | sch07_4221283 | 2.5 | 3.1 | -4.0 |
|  |  | 9 | 7.2 - 7.52 | ich09_7197158 | sch09_7521988 | 3.3 | 4.1 | -4.5 |
|  |  | 9 | **19.29 - 19.7** | **sch09_19288183** | **sch09_19699833** | 8.5 | 11.3 | -7.8 |
|  | SLRI | 1 | **4.05 - 4.67** | **01id_4052916** | **01id_4673121** | 2.9 | 4.5 | -3.8 |
|  |  | 2 | 21.33 - 21.38 | sch02_21334899 | sch02_21380162 | 3.8 | 5.6 | -4.2 |
|  |  | 5 | **20.41 - 23.05** | **05id_20410437** | **05id_23047412** | 10.0 | 16.5 | 7.2 |
|  |  | 7 | 14.51 - 14.61 | sch07_14510128 | ich07_14614284 | 6.5 | 9.9 | -5.5 |
|  |  | 9 | **19.29 - 19.7** | **sch09_19288183** | **sch09_19699833** | 11.1 | 18.2 | -7.8 |

^a^Chr., chromosome; LOD, logarithm of odds; PVE, phenotypic variance explained by QTL; add, additive effect.
Negative and positive additive effects mean that each allelic effect was derived from T887 and M23, respectively.
^b^Bold letters indicate significant loci affecting FLRI and SLRI in both field tests.

**Supplementary Table S6. Analysis of variance (ANOVA) of the agronomic traits of NILs and each recurrent parent.**

|  | Agronomic traits^a^ | | | |
| --- | --- | --- | --- | --- |
| Genotype | Panicle number | Panicle dry weight (g) | Plant dry weight (g) | Total dry weight (g) |
| T887 | 8 ± 1.1 | 18.4 ± 3 | 14.1 ± 2.5 | 32.5 ± 3.7 |
| T887-*qARO9*^M23^ | 8.3 ± 0.6 | 16.4 ± 1.3 | 13.9 ± 2.7 | 30.3 ± 3.4 |
| T887-*qARO1*^M23^+*qARO9*^M23^ | 8.7 ± 1.5 | 18.4 ± 4.7 | 13.5 ± 1.7 | 31.8 ± 6 |
| ANOVA | ns | ns | ns | ns |
|  |  |  |  |  |
| M23 | 7.4 ± 1.5 | 27.6 ± 5.4 | 22.2 ± 4.3 | 49.7 ± 9.5 |
| M23-*qARO9*^T887^ | 8 ± 1 | 30.4 ± 3.2 | 21.1 ± 4.5 | 51.5 ± 4.7 |
| M23-*qARO1*^T887^+*qARO9*^T887^ | 8.2 ± 0.8 | 24 ± 2.4 | 24.8 ± 2.6 | 47.7 ± 5 |
| ANOVA | ns | ns | ns | ns |

^a^Data represent mean ± standard deviation (SD). ns, non-significant.
